# G**α**_11_ deficiency increases fibroblast growth factor 23 levels in a mouse model of familial hypocalciuric hypercalcemia

**DOI:** 10.1172/jci.insight.178993

**Published:** 2024-03-26

**Authors:** Birol Ay, Sajin Marcus Cyr, Kaitlin Klovdahl, Wen Zhou, Christina M. Tognoni, Yorihiro Iwasaki, Eugene P Rhee, Alpaslan Dedeoglu, Petra Simic, Murat Bastepe

**Affiliations:** 1 Endocrine Unit, Department of Medicine, and; 2 Nephrology Division, Massachusetts General Hospital, Harvard Medical School, Boston, Massachusetts, USA.; 3 Department of Veterans Affairs, VA Boston Healthcare System, Boston, Massachusetts, USA.; 4 Department of Neurology, Boston University School of Medicine, Boston, Massachusetts, USA.; 5 Department of Radiology, Massachusetts General Hospital and Harvard Medical School, Massachusetts, USA.

**Keywords:** Endocrinology, G proteins, Genetic diseases, Mouse models

## Abstract

Fibroblast growth factor 23 (FGF23) production has recently been shown to increase downstream of Gα_q/11_-PKC signaling in osteocytes. Inactivating mutations in the gene encoding Gα_11_ (*GNA11*) cause familial hypocalciuric hypercalcemia (FHH) due to impaired calcium-sensing receptor signaling. We explored the effect of Gα_11_ deficiency on FGF23 production in mice with heterozygous (*Gna11*^+/–^) or homozygous (*Gna11*^–/–^) ablation of *Gna11*. Both *Gna11*^+/–^ and *Gna11*^–/–^ mice demonstrated hypercalcemia and mildly raised parathyroid hormone levels, consistent with FHH. Strikingly, these mice also displayed increased serum levels of total and intact FGF23 and hypophosphatemia. *Gna11*^–/–^ mice showed augmented *Fgf23* mRNA levels in the liver and heart, but not in bone or bone marrow, and also showed evidence of systemic inflammation with elevated serum IL-1β levels. *Furin* gene expression was significantly increased in the *Gna11*^–/–^ liver, suggesting enhanced FGF23 cleavage despite the observed rise in circulating intact FGF23 levels. *Gna11*^–/–^ mice had normal renal function and reduced serum levels of glycerol-3-phosphate, excluding kidney injury as the primary cause of elevated intact FGF23 levels. Thus, Gα_11_ ablation caused systemic inflammation and excess serum FGF23 in mice, suggesting that patients with FHH — at least those with *GNA11* mutations — may be at risk for these complications.

## Abstract 2

Loss of G protein alpha-subunit G⍺11, which mediating calcium-sensing receptor actions, increases the serum level of the phosphaturic hormone FGF23.

## Graphical abstract

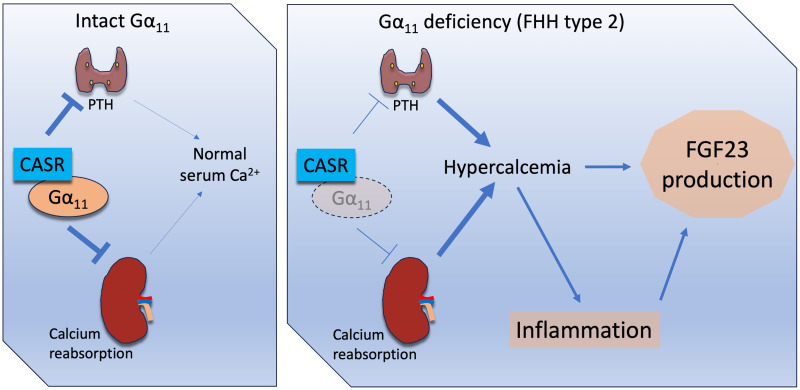

## Introduction

Fibroblast growth factor 23 (FGF23) is a bone-derived phosphaturic polypeptide hormone that regulates the renal handling of phosphate and vitamin D metabolism (1, 2). Mainly produced by mature osteoblasts and osteocytes (3, 4), FGF23 promotes renal phosphate excretion by inhibiting the reabsorption of phosphate from the glomerular filtrate and reduces the renal synthesis of the bioactive vitamin D metabolite, 1,25-dihydroxyvitamin D (1,25-[OH]_2_D) (5). While deficiency or impaired actions of FGF23 cause hyperphosphatemia and soft tissue mineralization (ectopic calcification), elevated FGF23 levels result in renal phosphate wasting and impaired skeletal mineralization (6, 7). Furthermore, increased serum levels of FGF23 are associated with a greater risk of left ventricular hypertrophy (8), heart failure (9), and atrial fibrillation (10) and are also independently associated with increased mortality in chronic kidney disease (CKD). Patients with acute kidney injury (AKI) also display elevated FGF23 levels, which are associated with an increased mortality (11) and risk of progression to CKD (12).

The major stimulators of skeletal FGF23 production include phosphate and 1,25(OH)_2_D, although several other factors have also been shown to promote skeletal FGF23 synthesis, including calcium and the parathyroid hormone (PTH) (13–15). Renal injury — as well as iron deficiency, inflammation, and anemia, which are often found in patients with CKD — also result in increased FGF23 production in bone (4, 16). Recently, glycerol-3-phosphate (G-3-P) has emerged as a kidney-derived factor that can mediate the effect of phosphate and ischemic kidney injury on skeletal FGF23 production. In renal injury or inflammation, FGF23 can also be synthesized in the bone marrow and other tissues, such as the spleen, liver, and heart (17–19). Therefore, the major contributing tissue to FGF23 production differs depending on the stimuli.

FGF23 is subject to posttranslational proteolytic cleavage, which converts the full-length bioactive intact peptide (iFGF23) into N-terminal and C-terminal fragments (20). The proteolytic cleavage and the resultant inactivation of FGF23 are crucial for phosphate homeostasis. Dysregulation of FGF23 cleavage results in excess or insufficient iFGF23 levels and is responsible for several human diseases, such as autosomal dominant hypophosphatemic rickets (7). PTH, inflammation, and iron deficiency increase not only the synthesis but also the cleavage of FGF23, thus resulting in elevated serum levels of total FGF23 (measured by using the C-terminal assay; cFGF23) without increasing iFGF23 (21–23). In contrast, phosphate, 1,25(OH)_2_D, and calcium promote skeletal FGF23 production without enhancing its cleavage, thereby leading to elevated levels of serum iFGF23 (24–28). Chronic kidney failure is also associated with suppressed FGF23 cleavage and, thus, increases serum levels of iFGF23 (29).

Multiple signaling pathways have been shown to mediate FGF23 production (30, 31). Two recent studies have indicated that protein kinase C (PKC) is among the mediators of FGF23 synthesis in osteoblasts and osteocytes (32, 33). In one of these studies, PKC activation and FGF23 synthesis appeared to be downstream of an as-yet-unidentified receptor that couples to the α subunits of the heterotrimeric G protein G_q/11_ — i.e., Gα_q_ and Gα_11_ (33). These ubiquitously expressed signaling proteins mediate the signaling of many heptahelical receptors, including the calcium-sensing receptor (CASR).

Heterozygous inactivating mutations in the gene encoding Gα_11_ (*GNA11*) cause familial hypocalciuric hypercalcemia (FHH), an autosomal dominant disorder characterized by persistently increased serum calcium levels, low urinary calcium, and inappropriately normal or raised PTH concentrations (34–37). These findings are due to impaired CASR signaling that primarily occurs in the kidney and the parathyroid gland, where serum calcium level is regulated through calcium reabsorption in the distal nephron and the synthesis and secretion of PTH, respectively. Some of the biochemical alterations in FHH, including increased calcium and PTH, can stimulate FGF23 production, while Gα_11_ deficiency may impair skeletal FGF23 production (32, 33). However, the levels of FGF23 have not been described in patients with loss-of-function *GNA11* mutations. In this study, we studied mice with homozygous (*Gna11*^–/–^) and heterozygous (*Gna11*^+/–^) global ablation of *Gna11* (a model of FHH type 2) to explore the effect of global Gα_11_ deficiency on FGF23 production.

## Results

### Gna11 ablation in mice phenocopies FHH and reveals elevated FGF23 levels.

**Figure 1:**
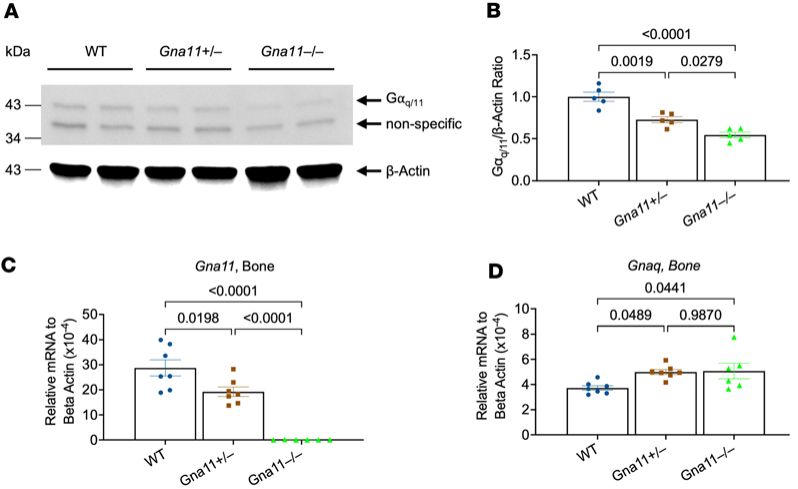
Gα_q/11_ protein levels and the gene expressions of *Gna11* and *Gnaq* in the femurs of *Gna11*-KO and WT mice. (**A**) A representative Western blot of Gα_q/11_ protein. (**B**) Densitometric quantification of Gα_q/11_ Western blots by ImageJ (NIH) (*n* = 5 mice/group). (**C** and **D**) The mRNA levels of (**C**) *Gna11* and (**D**) *Gnaq* genes (*n* = 7 mice/group) in the femurs from 2-month-old mice. β-Actin was used as a loading control in Western blots. (**B**–**D**) One-way ANOVA, followed by Tukey’s multiple comparisons, was used; data are shown as mean ± SEM.

Gα_q_ and Gα_11_ are functionally redundant proteins, but their relative abundance differs in individual tissues (38). To assess the relative expression levels of these proteins in bone, we used an antibody that recognizes both of these proteins indistinguishably and determined the effect of *Gna11* gene ablation on their total level (39). In femoral bone lysates, Western blots demonstrated that protein abundance of Gα_q/11_ was 72% ± 3.6% or 54% ± 3.4% of WT in *Gna11*^+/–^ and *Gna11*^–/–^, respectively (Figure 1, A and B), suggesting that Gα_q_ and Gα_11_ proteins are expressed at comparable levels. We also determined the alterations of *Gna11* and *Gnaq* mRNA levels in femurs from the mutant and WT littermates. As expected, while *Gna11* expression was 67% ± 9.8% of WT in *Gna11*^+/–^ mice, there was no considerable *Gna11* expression in *Gna11*^–/–^ mice (Figure 1C). The skeletal *Gnaq* mRNA level in *Gna11*^+/–^ and *Gna11*^–/–^ mice was not substantially different from that in WT mice, although a mild elevation of *Gnaq* mRNA level existed in each mutant mouse model relative to WT (Figure 1D). These findings indicate that skeletal Gα_q/11_ signaling may be partially, but not completely, impaired in these mutant mice.

**Figure 2:**
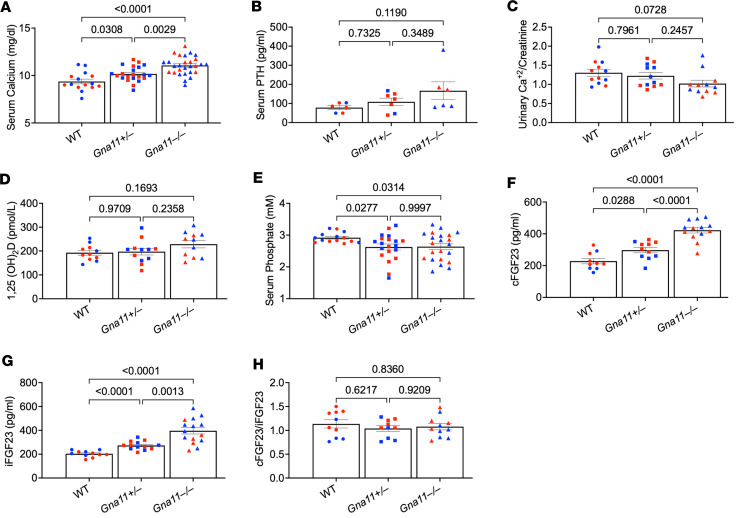
Serum and urine parameters of *Gna11*-KO and WT mice. (**A**–**H**) Serum calcium (*n* = 15–28 mice/group), parathyroid hormone (PTH) (*n* = 6–7 mice/group), urinary calcium/creatinine (*n* = 12 mice/group), 1,25-Dihydroxyvitamin D (1,25[OH]_2_D) (*n* = 12 mice/group), serum phosphate (*n* = 15–22 mice/group), total FGF23 (cFGF23) (*n* = 11–13 mice/group), intact FGF23 (iFGF23) (*n* = 11–15 mice/group), and cFGF23/iFGF23 ratio (*n* = 11 mice/group), demonstrating cleavage of FGF23. One-way ANOVA, followed by Tukey’s multiple comparisons, was used; data are shown as mean ± SEM. Blue, males; red, females.

Similar to the findings in patients with FHH, serum calcium levels were significantly elevated in *Gna11*^+/–^ and *Gna11*^–/–^ mice (Figure 2A), and serum PTH levels were inappropriately normal, with a trend for an increase in *Gna11*^+/–^ and *Gna11*^–/–^ mice (Figure 2B). The urinary calcium-to-creatinine ratio tended to be reduced in *Gna11*^–/–^ mice; however, the difference did not reach statistical significance (Figure 2C). Serum 1,25(OH)_2_D concentrations were comparable among the experimental groups (Figure 2D), whereas *Gna11*^+/–^ and *Gna11*^–/–^ mice displayed significantly reduced serum phosphate levels (Figure 2E). Strikingly, serum cFGF23 levels were significantly elevated in *Gna11*^+/–^ (1.3-fold) and *Gna11*^–/–^ (1.8-fold) mice compared with WT (Figure 2F). Similarly, serum iFGF23 levels were also significantly higher in *Gna11*^+/–^ (1.3-fold) and *Gna11*^–/–^ (1.9-fold) mice than in WT mice (Figure 2G). The cFGF23/iFGF23 ratio was comparable among the groups, indicating that the degree of FGF23 cleavage was not altered (Figure 2H). There were no sex-specific changes in the trends of the serum parameters.

### Fgf23 mRNA levels are elevated in the liver and heart, but not in the bone and bone marrow, of Gna11^–/–^ mice compared with WT littermates.

**Figure 3:**
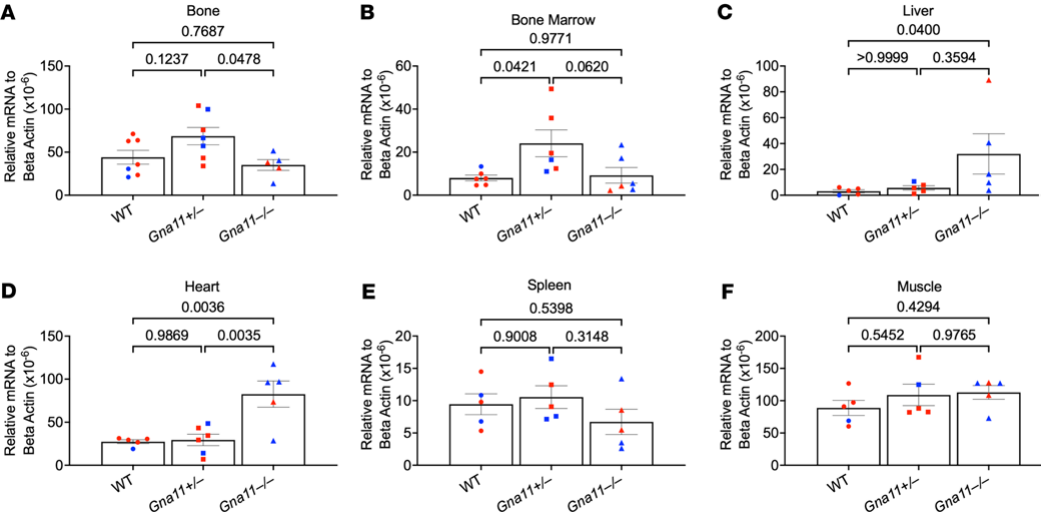
*Fgf23* gene expression in various tissues of *Gna11*-KO and WT mice. (**A**–**F**) *Fgf23* gene expression in bone (*n* = 5–7 mice/group), bone marrow (*n* = 6 mice/group), liver (*n* = 5 mice/group), heart (*n* = 5-6 mice/group), spleen (*n* = 5 mice/group), and muscle (*n* = 5 mice/group). (**A**, **B**, and **D**–**F**) One-way ANOVA, followed by Tukey’s multiple comparisons, was used. (**C**) Kruskal-Wallis, followed by Dunn’s multiple comparisons, was used. Data are shown as mean ± SEM. Blue, males; red, females.

To determine the tissue source of elevated FGF23 levels in *Gna11*^+/–^ and *Gna11*^–^^/–^ mice, we first measured *Fgf23* mRNA levels in bone and bone marrow, the 2 most substantial contributors to FGF23 production under many circumstances. In bone, the *Fgf23* mRNA level tended to be elevated (Figure 3A), and in bone marrow, it was significantly higher in *Gna11*^+/–^ mice (Figure 3, A and B). In contrast, *Gna11*^–/–^ mice displayed levels that are comparable with WT levels (Figure 3, A and B). Remarkably, *Fgf23* mRNA levels were significantly elevated in the liver (10-fold) and heart (3-fold) of *Gna11*^–/–^ compared with *Gna11*^+/–^ and WT (Figure 3, C and D), while no differences were detected in the muscle and spleen (Figure 3, E and F).

### Increased FGF23 production in Gna11^–/–^, but not in Gna11^+/–^, mice is associated with mild inflammation.

**Figure 4:**
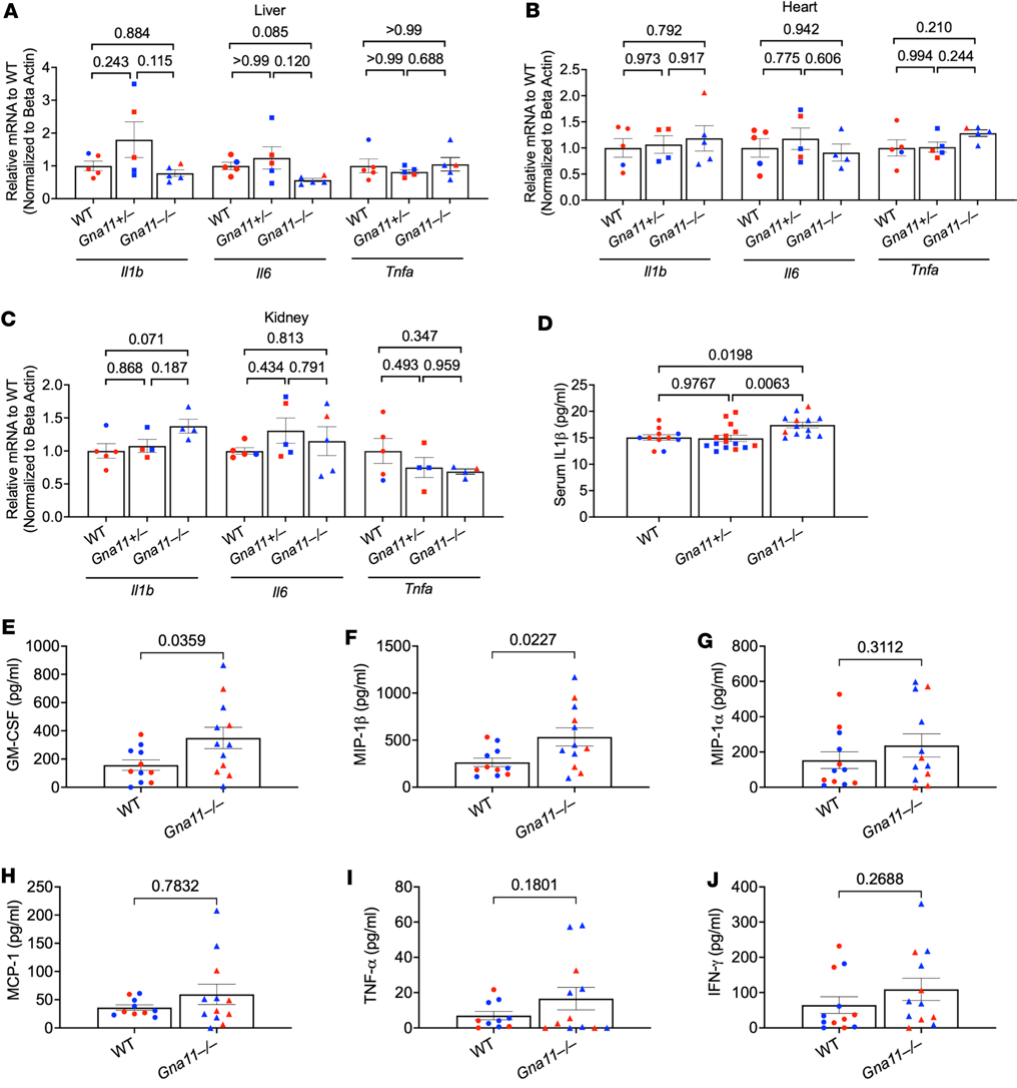
Inflammatory parameters in the serum, heart, kidney, and liver of *Gna11-*KO mice. (**A**–**C**) Gene expressions of *Il1b*, *Il6*, and *Tnfa* in liver, heart, and kidney (*n* = 4-5 mice/group) of *Gna11* KO mice. (**D**) Serum levels of IL-1β measured by ELISA (*n* = 11–15 mice/group). (**E**–**J**) Serum concentrations of GM-CSF, macrophage inflammatory protein-1 β (MIP-1β), macrophage inflammatory protein-1 α (MIP-1α), monocyte chemoattractant protein-1 (MCP-1), TNF-α, and IFN-γ detected by Luminex 200. (**A**) *Il6* and *Tnfa*: Kruskal-Wallis followed by Dunn’s multiple comparisons; all other 3 group comparisons in **A**–**C** used 1-way ANOVA followed by Tukey’s multiple comparisons. (**E**–**J**) *n* = 11–12 mice/group; 2-tailed Student’s *t* test. Data are shown as mean ± SEM. Blue, males; red, females.

Given that extraskeletal organs, such as the liver and heart, can produce FGF23 in inflammation, the increased *Fgf23* gene expressions in the liver and heart of *Gna11*^–/–^ mice suggested a possible role for inflammation. Nonetheless, expression of proinflammatory cytokine genes (*Il1b*, *Il6*, and *Tnfa*) was not significantly elevated in these tissues in both *Gna11*^+/–^ and *Gna11^–/–^* mice (Figure 4, A and B). Since renal injury, with or without inflammation, can also lead to extraskeletal FGF23 synthesis, we then analyzed the kidney. Quantitative PCR (qPCR) experiments revealed modestly increased *Il1b* mRNA levels in *Gna11*^–/–^ mice (1.3-fold) compared with *Gna11*^+/–^ and WT mice, although statistical significance was not reached (Figure 4C). Circulating IL-1β levels were also significantly, albeit mildly, higher in *Gna11*^–/–^ mice than in *Gna11*^+/–^ and WT mice (Figure 4D). Therefore, a further analysis of the inflammatory profile was only performed for *Gna11*^–/–^ mice. This analysis showed increased serum levels of granulocyte-macrophage–CSF (GM-CSF) and macrophage inflammatory protein-1 β (MIP-1β) in *Gna11*^–/–^ mice (Figure 4, E and F), while MIP-1α, monocyte chemoattractant protein-1 (MCP-1), TNF-α, and IFN-γ were not significantly higher (Figure 4, G–J). Aligned with the evidence of mild systemic inflammation, *Gna11*^–/–^ mice showed significantly increased hepatic expression levels of cytokine target genes, *Socs3* (2.6-fold, *P* = 0.019) and *Ikba* (1.5-fold, *P* = 0.011). On the other hand, *Gna11*^–/–^ liver had normal mRNA levels of *Errg*, and this was previously reported to be responsible for increased hepatic FGF23 production in an AKI mouse model (17) (Supplemental Figure 1; supplemental material available online with this article; https://doi.org/10.1172/jci.insight.178993DS1).

### Gna11^–/–^ mice do not have evidence of renal injury.

**Figure 5:**
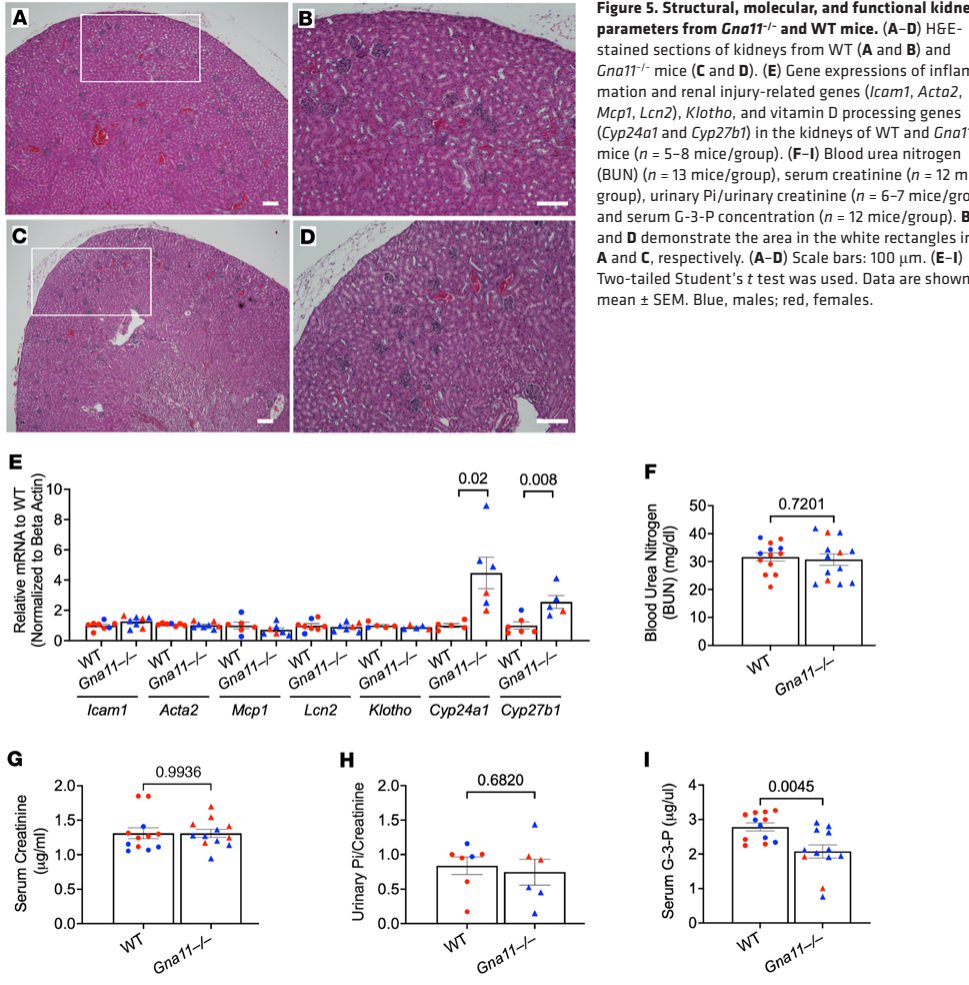
Structural, molecular, and functional kidney parameters from *Gna11*^–/–^ and WT mice. (**A**–**D**) H&E-stained sections of kidneys from WT (**A** and **B**) and *Gna11*^–/–^ mice (**C** and **D**). (**E**) Gene expressions of inflammation and renal injury-related genes (*Icam1*, *Acta2*, *Mcp1*, *Lcn2*), *Klotho*, and vitamin D processing genes (*Cyp24a1* and *Cyp27b1*) in the kidneys of WT and *Gna11*^–/–^ mice (*n* = 5–8 mice/group). (**F**–**I**) Blood urea nitrogen (BUN) (*n* = 13 mice/group), serum creatinine (*n* = 12 mice/group), urinary Pi/urinary creatinine (*n* = 6–7 mice/group), and serum G-3-P concentration (*n* = 12 mice/group). **B** and **D** demonstrate the area in the white rectangles in **A** and **C**, respectively. (**A**–**D**) Scale bars: 100 μm. (**E**–**I**) Two-tailed Student’s *t* test was used. Data are shown as mean ± SEM. Blue, males; red, females.

We examined renal histology, expression of fibrosis-related genes, and kidney functional parameters to determine whether the mild inflammation affected kidney structure and function. No remarkable histological differences were observed in H&E sections of kidneys extracted from WT (Figure 5, A and B) and *Gna11*^–/–^ (Figure 5, C and D) mice. In the gene expression analysis, markers of renal injury and inflammation, including *Icam1*, *Acta2*, and *Mcp1*, were not significantly altered (Figure 5E). The mRNA level of *Lcn2*, encoding the acute kidney injury marker neutrophil gelatinase-associated lipocalin (NGAL) (40, 41), was also comparable between *Gna11*^–/–^ and WT kidneys (Figure 5E)*.* The mRNA level of *Klotho*, which is necessary for the renal action of FGF23 (42) and which reduces during early stages of renal failure (43), was also unchanged (Figure 5E). Blood urea nitrogen (BUN) (Figure 5F), serum creatinine (Figure 5G), and urinary phosphate/urinary creatinine (Figure 5H) were comparable in *Gna11*^–/–^ and WT littermates, indicating that renal function is preserved. Recently, skeletal FGF23 production has been shown to increase in response to G-3-P, a glycolysis byproduct synthesized and secreted from the renal proximal tubule in response to phosphate and ischemic injury (44, 45). The circulating level of G-3-P was significantly lower in *Gna11*^–/–^ compared with WT littermates, consistent with the expected physiologic effect of decreased phosphate concentrations in *Gna11*^–/–^ mice (Figure 5I). While PTH is known to suppress *Cyp24a1* (24-hydroxylase encoding gene) and stimulate *Cyp27b1* (1α-hydroxylase encoding gene) to increase 1,25(OH)_2_D production, the effects of FGF23 on these genes are opposite (46). In our *Gna11*^–/–^ mice, where both PTH and FGF23 levels were elevated, the renal expression levels of both *Cyp24a1* (4.4-fold, *P* = 0.02) and *Cyp27b1* (2.5-fold, *P* = 0.0008) were significantly increased, likely reflecting the counteracting effects of these hormones (Figure 5E). These findings suggest that the high FGF23 levels in *Gna11*^–/–^ mice are unlikely to be secondary to kidney injury.

### Furin gene expression is increased in the liver of Gna11^–/–^ mice.

**Figure 6:**
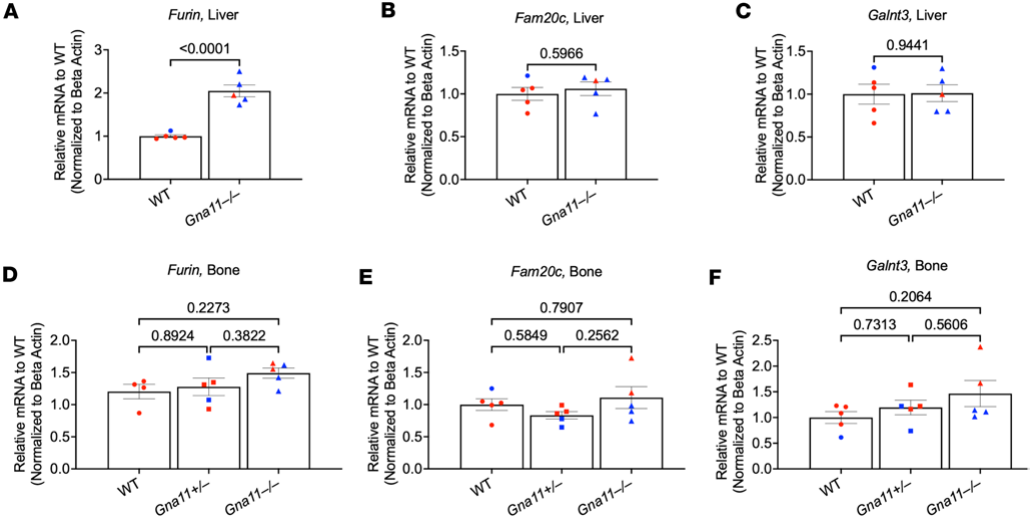
FGF23 processing gene expressions in the liver and bone of *Gna11*-KO and WT mice. (**A**–**C**) *Furin*, *Fam20c*, *Galnt3* gene expressions in the liver. (**D**–**F**) *Furin*, *Fam20c*, *Galnt3* gene expressions in bone. (**A**–**C**) Two-tailed Student’s *t* test was used. (**D**–**F**) One-way ANOVA, followed by Tukey’s multiple comparisons, was used. Data are shown as mean ± SEM. (**A**–**F**) *n* = 4–5 mice/group. Blue, males; red, females.

Since inflammation-induced FGF23 production is typically coupled with accelerated FGF23 cleavage (47, 48), we analyzed the hepatic expression levels of genes encoding FGF23 processing enzymes. While *Furin* expression in the liver was significantly elevated (2-fold) in *Gna11*^–/–^ compared with WT mice (Figure 6A), mRNA levels of *Fam20c* and *Galnt3* were not significantly altered (Figure 6, B and C), suggesting that the cleavage of FGF23 produced in the liver is increased. Thus, it is unlikely that the liver markedly contributes to the observed increase in the serum iFGF23 levels in *Gna11*^–/–^ mice.

Given that the skeletal *Galnt3* expression level critically regulates the production of iFGF23 in response to phosphate, seemingly in the absence of an increase in FGF23 mRNA levels (26), we asked if alterations in FGF23 cleavage in bone play a role in the observed elevation of serum iFGF23 in *Gna11*^–/–^ mice. No remarkable differences were detected in the mRNA levels of *Furin* (Figure 6D), *Fam20c* (Figure 6E), or *Galnt3* (Figure 6F) in the femurs of *Gna11*^+/–^, *Gna11*^–/–^, and WT groups, making it unlikely that the increased serum iFGF23 levels reflect diminished FGF23 cleavage in bone. Moreover, we measured cFGF23 and iFGF23 protein levels in femur lysates using ELISA, as described previously (49, 50), but we did not detect any significant differences among *Gna11*^+/–^, *Gna11*^–/–^, or WT littermates (Supplemental Figure 2, A and B). In contrast, using the same method, we could detect 7.8- and 6.6-fold elevations of cFGF23 and iFGF23 protein levels in the femur of an adenine-rich diet–induced CKD mouse model. Serum concentrations of cFGF23 and iFGF23 in this model, however, were 22-fold and 16-fold higher than in control mice, respectively (Supplemental Figure 2, C–F), suggesting that the FGF23 ELISAs have poor sensitivity when used in tissue lysates.

## Discussion

This study found that ablating *Gna11* in mice models the human FHH phenotype with elevated serum calcium and mildly increased serum PTH levels. Interestingly, cFGF23 and iFGF23 are elevated in both *Gna11*^+/–^ and *Gna11*^–/–^ mice. We also found evidence of systemic inflammation and extraskeletal FGF23 production in *Gna11*^–/–^ mice.

FHH results from disrupted signaling downstream of CASR, which can be due to heterozygous inactivating mutations in the *CASR* gene, *GNA11*, or *AP2S1* (36, 37). The CASR couples primarily to Gα_11_ and Gα_q_ proteins, which are functionally redundant. However, the relative levels of these proteins vary depending on the cell type, and therefore, in tissues where Gα_11_ is more abundant than Gα_q_, the loss of the former alone markedly attenuates receptor signaling. This event is considered the underlying cause of CASR signaling deficiency in patients carrying FHH with inactivating *GNA11* mutations. Displaying serum biochemistries like those observed in patients with FHH, our *Gna11*-KO mouse models probably show a similar disease mechanism.

Serum measurements of FGF23 have not been described for patients with FHH in the literature (34, 51, 52). However, mice with a missense *Ap2s1* mutant, which also phenocopied FHH, were recently shown to have increased serum intact FGF23 levels (53). Therefore, the elevated serum FGF23 levels in our mouse model may reflect the observed FHH-related systemic alterations. Notably, given that elevated serum FGF23 levels are directly associated with cardiovascular morbidity in patients with or without renal disease (54, 55), monitoring serum FGF23 levels may be helpful in patients with FHH to assess the risk of cardiovascular diseases.

In addition to increased serum IL-1β levels, *Gna11*^–/–^ mice display elevated serum GM-CSF and MIP-1β, further indicating macrophage activation and proinflammatory phase (Figure 4, E and F). Increased calcium concentrations can stimulate inflammasomes, resulting in IL-1β production from bone marrow–derived macrophages by CASR signaling, which relies, in this setting, primarily on the Gsα protein (56). Therefore, the elevated calcium and the activation of CASR in macrophages may be responsible for the increased serum IL-1β levels in our *Gna11*^–/–^ mice. Moreover, recent studies suggest that FGF23 exerts proinflammatory actions (57, 58), making it possible that the elevated FGF23 also contributes to systemic inflammation in these mice.

Inflammation stimulates FGF23 production by increasing both *Fgf23* transcription and FGF23 cleavage, which increases serum cFGF23 excessively rather than serum iFGF23 concentrations. In recent studies, IL-1β injections, used to create inflammation in animal models, increased cFGF23 more than iFGF23, demonstrating increased cleavage in acute and chronic inflammation models (22, 58). Indeed, *Gna11*^–/–^ mice showed significantly increased *Furin* mRNA expression in the liver compared with WT, predicting increased cleavage of FGF23 protein produced in the liver of *Gna11*^–/–^ mice. Therefore, while the inflammation in *Gna11*^–/–^ mice could explain the elevation of *Fgf23* mRNA levels in extraskeletal tissues, it is unlikely to account for the increase of both total and iFGF23 in the serum, without evidence of increased cleavage (Figure 2H). Moreover, *Gna11*^+/–^ mice also displayed significantly elevated levels of serum cFGF23 and iFGF23 despite a lack of augmented *Fgf23* mRNA expression in extraskeletal tissues and inflammation. Thus, additional mechanisms are likely involved in the increased circulating iFGF23 levels in both *Gna11*^+/–^ and *Gna11*^–/–^ mice (Figure 2G).

Calcium is a crucial regulator of FGF23 production, and studies indicate that FGF23 synthesis is impaired in the setting of hypocalcemia (13, 59). Calcium also stimulates FGF23 synthesis, even without 1,25(OH)_2_D actions, increasing both *Fgf23* mRNA levels in bone and iFGF23 in serum (60). Thus, hypercalcemia may account for the elevated serum iFGF23 and skeletal *Fgf23* mRNA levels observed in *Gna11*^+/–^ mice. In contrast, *Gna11*^–/–^ mice showed no detectable elevation of skeletal *Fgf23* mRNA expression despite a more substantial increase in serum calcium. This finding could, perhaps, reflect a skeletal resistance to the effect of calcium due to the approximately 50% reduction we detected in the total amount of Gα_q_ and Gα_11_ proteins. This reduction may impair the signaling of CASR. However, the FGF23-inducing action of calcium in bone has been suggested to occur via L-type calcium channels, and no evidence for an involvement of CASR was presented (13). Therefore, the putative skeletal resistance to calcium regarding FGF23 production may reflect the impaired signaling of another Gα_q/11_-coupled receptor. Nevertheless, despite unchanged *Fgf23* mRNA levels in bone, *Gna11*^–/–^ mice showed increased serum iFGF23. The serum PTH levels tended to be elevated in *Gna11*^–/–^ mice; however, PTH-induced FGF23 production also entails increased *Fgf23* gene expression in bone and is coupled with enhanced cleavage (21), arguing against a role for PTH in the increased serum iFGF23 in *Gna11*^–/–^ mice.

It has been reported in clinical studies that hypercalcemia is associated with kidney injury (61–63), which is known to raise iFGF23 levels (64). However, although a mildly increased *Il1b* mRNA level was detected, serum BUN and creatinine levels — as well as the levels of multiple kidney fibrosis and injury markers, including *Lcn2* and *Klotho* mRNA — were comparable between WT and *Gna11*^–/–^ mice. We also did not observe an increase in the circulating level of G-3-P, a kidney injury–derived metabolite that stimulates skeletal FGF23 synthesis (44, 45). Thus, renal injury is unlikely to explain the elevated FGF23 levels in *Gna11*^–/–^ mice.

The significantly reduced phosphate levels in both *Gna11*^+/–^ and *Gna11*^–/–^ mice argue against a role for phosphate in the increased FGF23 production in these mice. The reduced levels of circulating G-3-P in *Gna11*^–/–^ mice are consistent with reduced phosphate-stimulated glycolysis in the kidney, which was recently identified as a phosphate sensor upstream of skeletal FGF23 production (45). Moreover, an important mechanism mediating phosphate-induced FGF23 production in bone is the increased *Galnt3* expression level (26), and we did not detect significant changes in the skeletal expression levels of FGF23 processing genes, including *Galnt3* in *Gna11*^+/–^ and *Gna11*^–/–^ mice. Interestingly, the skeletal expression level of *Furin* is also unchanged in *Gna11*^–/–^ mice despite the increased serum IL-1β levels, as opposed to the findings of a recent study showing a time-dependent increase of skeletal *Furin* expression in response to a single IL-1β injection (58). The lack of an increased *Furin* level in our model may be due to the mildness of IL-1β elevation.

In a cohort of healthy children, a positive correlation between iFGF23 levels and serum calcium and a negative correlation between iFGF23 levels and urinary calcium/creatinine ratio have been demonstrated (65). Moreover, in FGF23-KO mice, renal calcium reabsorption and renal membrane abundance of TRPV5 (an epithelial calcium channel) were reduced, demonstrating the role of FGF23 as a calcium-conserving hormone in the kidney (66). Therefore, increased serum FGF23 levels are expected to stimulate renal calcium reabsorption and further exacerbate hypercalcemia in our *Gna11*-KO mice.

One of the limitations of our study is that we cannot pinpoint the tissue source of elevated serum iFGF23 in *Gna11*^+/–^ and *Gna11*^–/–^ mice. No significant differences existed between *Gna11*-KO and WT mice in the skeletal levels of the FGF23 protein, even though we used a sensitive detection method, FGF23 ELISA (50), which could reveal a 6.6-fold increase in the bone of an adenine-rich diet–induced CKD mouse model compared with control mice. However, the degree of serum FGF23 elevation in *Gna11*-KO mice was modest compared with the CKD model, in which serum cFGF23 and iFGF23 levels were elevated 22-fold and 16-fold compared with controls, respectively (Supplemental Figure 2, A–D). At least 2 other studies used the same FGF23 ELISA for quantifying tissue levels of FGF23 protein. They successfully measured the elevation of FGF23 protein amount in a polymicrobial sepsis model or an LPS-induced sustained inflammation model, where the elevation was 6-fold in the bone (50) and 15-fold in the spleen (18), respectively. Like our findings, however, the elevations of serum FGF23 levels observed in these other disease models were markedly greater than what was detected in tissue lysates (18, 50). While the higher elevation in the serum compared with the analyzed tissues may reflect FGF23 production from additional tissues, it is plausible that the sensitivity of the FGF23 ELISA is substantially higher for serum than tissue lysate measurements. Thus, we cannot entirely rule out the possibility that FGF23 protein is made in the bones of *Gna11*-KO mice.

In conclusion, the murine phenotype of *Gna11* ablation resembles FHH but additionally includes elevated FGF23 levels and mild systemic inflammation. Based on our findings, patients with FHH may display increased FGF23 synthesis, which could contribute to the clinical phenotype.

## Methods

### Sex as a biological variable.

Our study examined male and female animals, and similar findings are reported for both sexes.

### Mice.

*Gna11*-KO mice were donated by Stefan Offermanns (Max Planck Institute for Heart and Lung Research, Bad Nauheim, Germany), and the ablation of *Gna11* in mice has been described previously (67). Both male and female homozygous (*Gna11*^–/–^) and heterozygous (*Gna11*^+/–^) *Gna11*-KO mice and age-matched WT (*Gna11^+/+^)* littermates were maintained in the C57BL/6 background. The analyses were performed at the age of 2 months. Mice were housed on a 12-hour light/12-hour dark cycle and were given access to water and a regular chow diet containing 1.09% calcium, 0.79% phosphorus, and 2.5 IU vitamin D_3_/g (RMH 3000, Prolab). Minimum sample size was determined by a power analysis considering standardized effect size (i.e., the difference between means divided by the standard deviation), which was estimated by preliminary measurements for each experiment. In addition, independent samples were collected from multiple litters. Therefore, sample sizes among different experiments varied. Fifteen-month-old WT male mice (C57BL/6J, The Jackson Laboratory) were fed an adenine-rich diet (0.2% adenine) for 4 weeks to induce chronic renal injury, as described (68), to demonstrate the elevation of FGF23 protein levels in serum and femurs with FGF23 ELISA kits.

### Tissue collection.

Animals were sacrificed by cervical dislocation following CO_2_ exposure. The liver, heart, spleen, kidney, and muscle were extracted and snap-frozen in liquid nitrogen. Femurs were removed and cleaned off surrounding muscle tissue, and bone marrow was separated with a quick spin after cutting the proximal and distal ends of the femurs. Both femurs and bone marrow were then snap-frozen in liquid nitrogen and kept at –80°C until use for gene expression and protein analysis. Kidneys were preserved in 10% neutral buffered formalin at 4°C overnight and transferred into 70% ethanol for histological analysis.

### Histology.

Tissue processing and H&E staining of formalin-fixed kidneys were performed at the MGH Center for Musculoskeletal Research, Histology & Histomorphometry Core. H&E-stained sections of kidneys were imaged with an all-in-one Keyence microscope (BZ-X, Keyence).

### Serum and urine biochemical parameters.

Under anesthesia with 3% isoflurane, blood was collected from the retroorbital vein into the heparin blood collection tubes to prepare serum. Urine was also collected just before anesthesia. Serum cFGF23 (60-6300, Quidel); iFGF23 (60-6800, Quidel); PTH 1-84 (60-2305, Quidel); 1,25(OH)_2_D (AC-62F1, IDS); and IL-1β (MLB00C, R&D Systems) were measured using ELISA kits according to the manufacturer`s instructions. Total calcium (procedure no. 0150, Stanbio) in serums and urines, and BUN (Procedure No. 2020, Stanbio) in serums, were measured as indicated in the kits’ manuals. Serum and urine phosphate were measured spectroscopically using a colorimetric phosphate assay kit (ab65622, Abcam). Urine creatinine was assessed with a direct creatinine assay kit from Stanbio (procedure no. 0430). Absorbance readings were obtained from an Envision microplate reader (PerkinElmer). Serum creatinine and G-3-P levels were measured from 10 μL of serum using liquid chromatography–mass spectrometry (LC-MS) as previously described (45). Serum cytokines (GM-CSF, MIP-1β, MIP-1α, MCP-1, TNF-α, and IFN-γ) were measured with a Milliplex assay (MCYTOMAG-70K, MilliporeSigma) using Luminex 200 system (40-012, MilliporeSigma) following the manufacturer’s instructions.

### Measurement and normalization of FGF23 proteins from tissues.

Total protein extracts were prepared by crushing and homogenizing the frozen tissue samples by motorized tissue grinder (Thermo Fisher Scientific) in RIPA lysis buffer (89900, Thermo Fisher Scientific) containing cOmplete Protease Inhibitor Cocktail tablets (Roche). Protein extracts were then centrifuged (13,000*g* for 10 minutes at 4°C) to remove nonhomogenized tissue parts, and the supernatant was collected into new 1.5 mL tubes. Extracts were diluted in a 1:1 ratio with the ELISA Kit standard 1 (0 pg/mL iFGF23 or cFGF23; Quidel) before measuring FGF23 protein amounts with the Quidel ELISA kits (cFGF23, 60-6300; iFGF23, 60-6800). FGF23 levels were normalized to the total protein amounts measured from each sample using the Pierce BCA protein assay kit (Thermo Fisher Scientific) (49, 50).

### qPCR.

Total RNA was extracted from the tissues using the RNeasy Mini Kit (74104, Qiagen) and converted into cDNA using a first-strand synthesis kit (E6560, New England BioLabs). cDNAs used to detect *Fgf23* gene expression were prepared with oligo(dT) and a gene-specific primer (5′-GTAGACGTCATAGCCATTC-3′) at a 1:1 ratio to improve the detection of *Fgf23* gene expression. TaqMan MGB probes (*Fgf23*, Mm00437132_m1; β*-actin*, Mm00607939_s1) along with TaqMan Fast Advanced Master Mix (4444557, Applied Biosystems) were used to quantify *Fgf23* mRNA levels. SYBR Green qPCR Master Mix (A25742, Applied Biosystems) was employed for all other gene expressions according to the manufacturer’s directions. Primer sequences used with SYBR green are provided in Supplemental Table 1. All qPCR reactions were run in an Applied Biosystems QuantStudio 3D Digital PCR System (Applied Biosystems).

### Immunoblotting.

Femurs were lysed using RIPA lysis buffer (89900, Thermo Fisher Scientific) containing cOmplete protease inhibitor cocktail tablets (Roche). The samples were centrifuged at 13,000*g* for 10 minutes at 4°C, and the supernatants were collected. Lysates were then separated by 7.5% SDS-PAGE, and the proteins were transferred to nitrocellulose membranes (Bio-Rad). Western blots were blocked with 5% milk in TBST and incubated with Gα_q/11_ (F5, sc-515689, Santa Cruz Biotechnology Inc.) antibody. The following day, blots were washed and incubated with the appropriate HRP-coupled secondary antibody, and the signals were detected with ECL (Pierce, Thermo Fisher Scientific). ImageJ (version 1.54f; NIH) was used for densitometric quantification of the blots. β-Actin (C4, sc-4778, Santa Cruz Biotechnology Inc.) antibody was used as the loading control.

### Statistics.

Data are presented as mean ± SEM as individual data points. Unpaired 2-tailed Student’s *t* test was utilized to test the significance of the difference between 2 groups. Welch’s correction was applied if the variances were significantly different, as determined by the F test. One-way ANOVA with Tukey’s post hoc test or the nonparametric equivalent, Kruskal-Wallis with Dunn’s post hoc test, was performed, based on whether the data were normally distributed, to assess the statistical significance of differences among WT, *Gna11*^+/–^, and *Gna11*^–/–^ mice as indicated in the figure legends. Single outliers in individual data sets, if any, were identified by the Grubbs’ test and excluded before statistical analyses. *P* < 0.05 was considered significant. Analyses were performed using GraphPad Prism (version 9.5.1).

### Study approval.

All animal experiments complied with the *Guide for the Care and Use of Laboratory Animals* (National Academies Press, 2011) and were approved by the research animal care committee at Massachusetts General Hospital and Harvard Medical School. All methods comply with the ARRIVE guidelines (69).

### Data availability.

Values for all data points in graphs are reported in the Supporting Data Values file.

## Author contributions

BA, MB, PS, YI, EPR, and AD designed the experiments and interpreted the data. SMC and KK managed the mouse colonies and genotyped the mice. BA performed Western blot, qPCR, ELISA, and serum/urine collection and measurements. WZ measured serum G-3-P and creatinine by HPLC. CMT detected serum cytokine concentrations by Luminex. MB and BA wrote the manuscript with input from all authors.

## Supplementary Material

Supplemental data

Unedited blot and gel images

Supporting data values

## Version 1 03/26/2024

In-Press Preview

## Version 2 05/08/2024

Electronic publication
