# Supplementary material for: Gα11 deficiency increases fibroblast growth factor 23 levels in a mouse model of familial hypocalciuric hypercalcemia
Source: JCI Insight. 2024 Mar 26;9(9):e178993. doi: 10.1172/jci.insight.178993 (PMC11141917; doi:10.1172/jci.insight.178993)
Supplement: Supplemental data [file jciinsight-9-178993-s146.pdf]

**Supplementary Information**

# $G\alpha_{11}$ deficiency increases fibroblast growth factor-23 levels in a mouse model of Familial Hypocalciuric Hypercalcemia

**B. Ay et al.**

**Supplementary Table S1.** Sequences of primer pairs used in qRT-PCR.

| Gene | Forward (5'-> 3') | Reverse (5'-> 3') |
|---|---|---|
| *Acta2* | TGAAGAGGAAGACAGCACAGC | GCCCATTCCAACCATTACTCC |
| *Cyp24a1* | GTGCGGATTTCCTTTGTGAT | GGGATTCCGGGATAGATTGT |
| *Cyp27b1* | CAAATGGCTTTGTCCCAGAT | GGCTGTCTTCCGAATGGTTA |
| *Errg* | AAGATCGACACATTGATTCCAGC | CATGGTTGAACTGTAACTCCCAC |
| *Fam20c* | GCTCATCATGACCTTCCAGA | CGCATTGTGCCTCTCATAGT |
| *Furin* | GGAGCCAGTTTTGACGTGAA | CTACGCCACAGACACCATTG |
| *Galnt3* | GAATTTCTCTGCACCGGGAC | CGTGGACCATGCTTCATTGT |
| *Gna11* | GGTCGATGTGGAGAAGGTCA | CCACGTCCGTCAAGTAGTACT |
| *Gnaq* | CAGACAATGAGAACCGCATG | TCGACTAGGTGGGAATACATGA |
| *Icam1* | CTGTTTGAGCTGAGCGAGAT | GCTCCACACTCTCCGGAAA |
| *Ikba* | GAAAGCTGGCTGTGATCCTG | GCGTCAAGACTGCTACACTG |
| *Il1b* | AGTTGACGGACCCCAAAAGA | GCTCTTGTTGATGTGCTGCT |
| *Il6* | TGAACAACGATGATGCACTTG | ACTCCAGAAGACCAGAGGAA |
| *Klotho* | AGACCTCCCGATGTATGTGAC | GTCCAACACGTAGGCTTTCA |
| *Lcn2* | CATTTGTTCCAAGCTCCAGGG | CATGGCGAACTGGTTGTAGTC |
| *Mcp1* | AGGTGTCCCAAAGAAGCTGT | GATCTCATTTGGTTCCGATCCA |
| *Socs3* | CCTCCTTTTCTTTGCCACCC | GCTCAGTACCAGCGGAATCT |
| *Tnfa* | CCACCACGCTCTTCTGTCTA | GGTCTGGGCCATAGAACTGA |
| *Actb* | GATCTGGCACCACACCTTCT | GGGGTGTTGAAGGTCTCAAA |

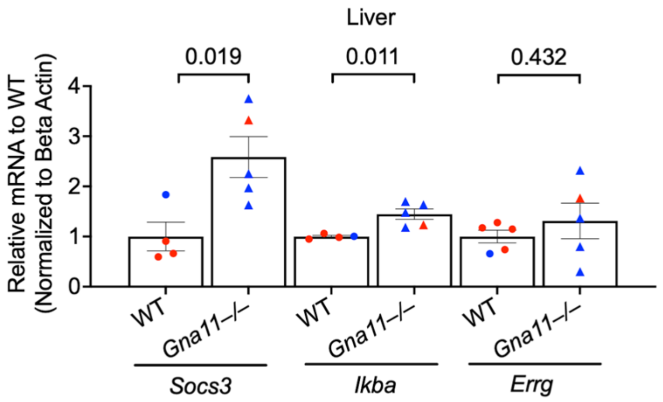


**Supplementary Figure 1. Expression levels of cytokine target genes, *Socs3, Ikba*, and *Errg,* in the liver of *Gna11*$^{-/-}$ mice.** Two-tailed Student`s t-test, mean ± SEM, n = 4-5 mice. Blue: Males, Red: Females.

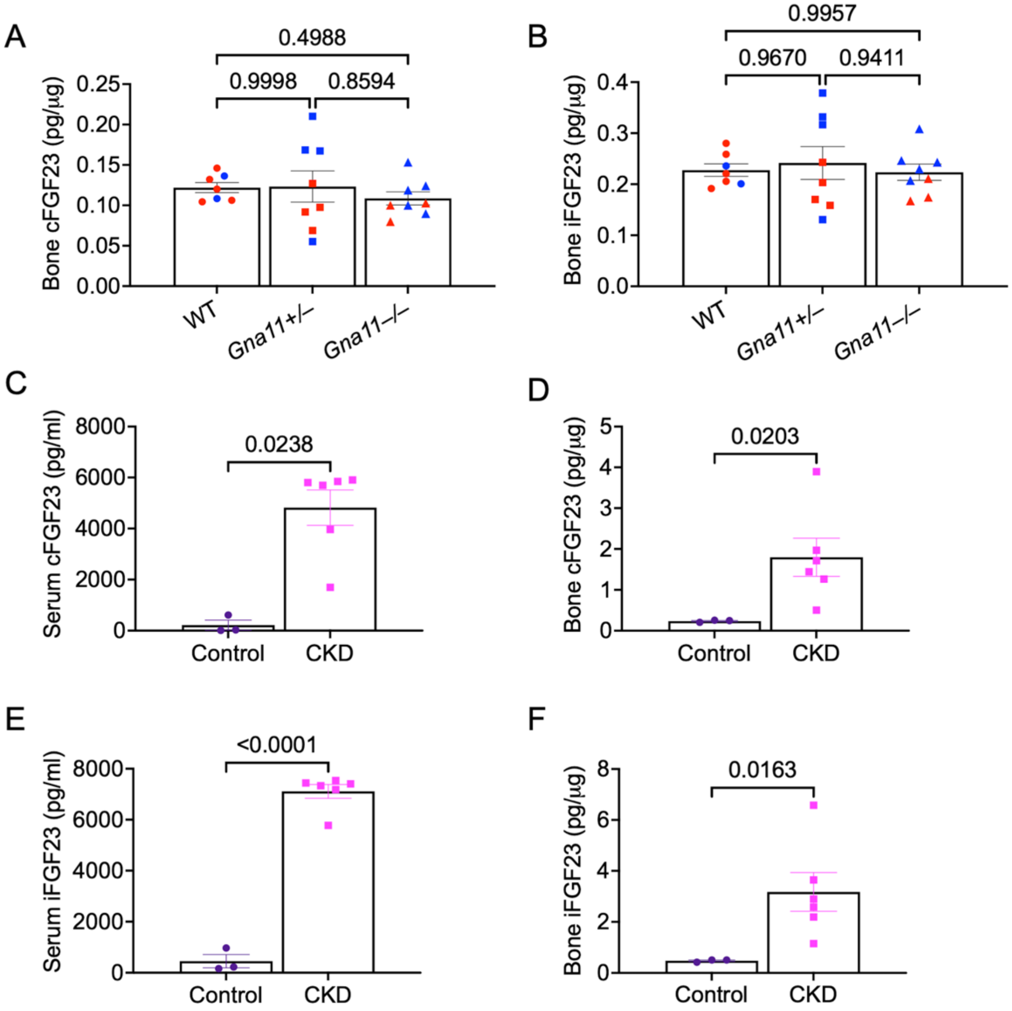


**Supplementary Figure 2. Bone FGF23 concentrations of *Gna11* KO mice along with serum and bone FGF23 measurements of CKD mice**. (**A**) Bone cFGF23 and (**B**) bone iFGF23 of *Gna11* KO mice, (**C**) Serum cFGF23, (**D**) bone cFGF23, (**E)** serum iFGF23, and (**F**) bone iFGF23 concentrations in CKD mice. A and B: One-Way ANOVA followed by Tukey`s multiple comparisons, mean ± SEM, n = 7-8 mice/group, Blue: Males, Red: Females, C-F: Two-tailed Student`s t-test, mean ± SEM, n = 3-6 mice/group.
