# Supplementary figures and images for: Gα11 deficiency increases fibroblast growth factor 23 levels in a mouse model of familial hypocalciuric hypercalcemia
Source: JCI Insight. 2024 Mar 26;9(9):e178993. doi: 10.1172/jci.insight.178993 (PMC11141917; doi:10.1172/jci.insight.178993)

Ay et al. - Full unedited gel for Figure 1A

Galphaq/11 antibody:

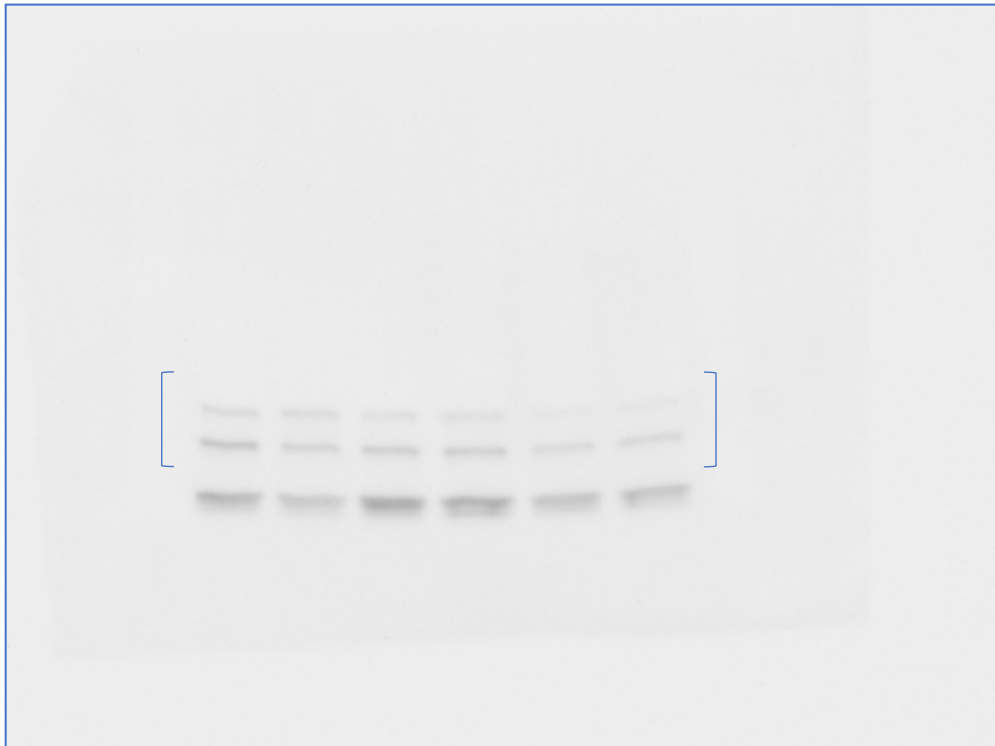

Beta-actin antibody blot:

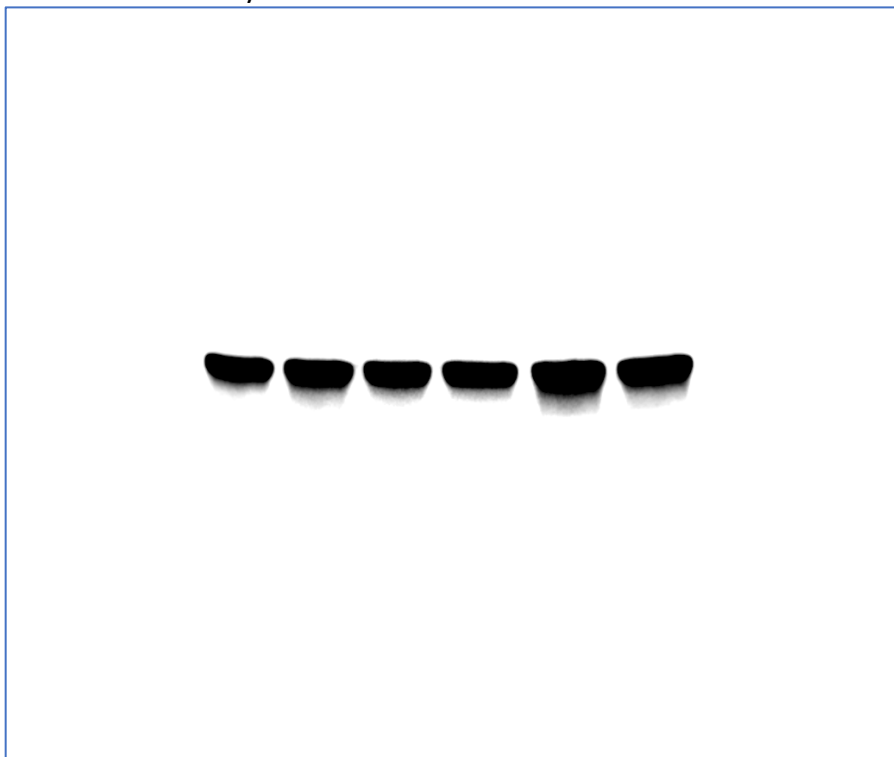

Supplement: Unedited blot and gel images [file jciinsight-9-178993-s147.pdf]
